# The global burden of low back pain attributable high body mass index over the period 1990–2021 and projections up to 2035

**DOI:** 10.3389/fnut.2025.1568015

**Published:** 2025-06-06

**Authors:** Jishi Ye, Jingli Chen, Huang Ding, Zhongyuan Xia, Juan Wang, Yifan Jia

**Affiliations:** ^1^Department of Pain, Renmin Hospital of Wuhan University, Wuhan, Hubei, China; ^2^Department of Anesthesiology, The Central Hospital of Wuhan, Tongji Medical College, Huazhong University of Science and Technology, Wuhan, Hubei, China

**Keywords:** low back pain, high body mass index, sociodemographic index, GBD, sex difference

## Abstract

**Objective:**

Low back pain (LBP) is a widely disease burden that transcends geographical, cultural, and demographic boundaries, with high body mass index (BMI) recognized as one of its well-established risk factors.

**Methods:**

Data on LBP attributed to high BMI were retrieved from the Global Burden of Disease (GBD) study 2021 online database. To evaluate the magnitude and direction of the trend in DALYs, a Joinpoint Regression Analysis (JRA) was performed. Additionally, an autoregressive integrated moving average (ARIMA) model was utilized to forecast future trends in DALYs indicators associated with LBP for the subsequent 15 years.

**Results:**

The impact of high BMI on LBP has shown a progressive increase annually from 1990 to 2021, with a more pronounced disease burden observed in regions with higher economic status. Within areas of comparable economic levels, older age correlates with a heightened burden of disease, and females experience a relatively greater disease burden compared to males. Projections based on the autoregressive integrated moving average (ARIMA) model indicate that the global burden of disease is predicted to continue its upward trajectory over the next 15 years.

**Conclusion:**

Higher levels of BMI, along with age and gender, are associated with an increased burden of LBP. The global burden of LBP attributable to high BMI is predicted to continue rising over the next 15 years. This study offers additional evidence to support the development and implementation of policies and strategies aimed at preventing and mitigating future increases in the burden of LBP.

## Highlights

- The findings indicate that the impact of high BMI on low back pain increased annually from 1990 to 2021.
- Regions with high sociodemographic index (SDI), indicative of economic development, exhibited a significantly greater burden of disease.
- The findings indicated that, within the same economic level, individuals of older age experience a greater burden of disease. Furthermore, within the same age group, females exhibit a higher burden of disease compared to males.
- The results showed that the global burden of low back pain (LBP) disease continues to rise, with the highest burden of disease in regions with high SDI, and the burden of LBP in females was significantly higher than that in males.

## Background

Low back pain (LBP) is a pervasive musculoskeletal symptom that transcends geographical, cultural, and demographic boundaries, affecting individuals across all age groups from childhood to the elderly (1, 2). It is acknowledged as a leading cause of disability worldwide, representing a significant healthcare challenge not only in developing countries but also in developed nations. The Global Burden of Disease (GBD) study indicates that LBP is a major contributor to years lived with disability (YLD), surpassing other chronic conditions such as diabetes (38.6 million), chronic obstructive pulmonary disease (30.6 million), and other diseases or conditions. This alarming trend reveals that LBP is responsible for around 64.9 million YLD globally (1). With the predicted rise in disability burden and healthcare costs associated with LBP, there is a pressing need for the development of effective management strategies and policies.

Given the high prevalence and substantial burden of LBP, it is imperative for researchers and healthcare policymakers to regularly update epidemiological data. This data serves as a critical tool in understanding the dynamics of LBP, assessing healthcare needs, and tailoring effective intervention strategies. The multifaceted nature of LBP extends beyond clinical manifestations; it incorporates various risk factors, thereby necessitating a comprehensive approach to its investigation and understanding.

In recent years, a plethora of studies have identified numerous risk factors associated with LBP, which can be categorized into biological, psychosocial, and behavioral factors. Key contributors include age, sex, education level, smoking status, high body mass index (BMI), and engaging in manual labor (3–5). Among these, high BMI is of particular concern. It is a commonly used metric for assessing an individual's weight relative to their height, primarily serving as an indicator of overweight and obesity. High BMI has been linked to an increased risk of several serious health issues, including cardiovascular diseases (6), type 2 diabetes (7), respiratory problems (8), arthritis (9), certain cancers (10), and mental health issues. The study revealed a strong correlation between high BMI and LBP, indicating that for every 5% increase in BMI, the risk of LBP increases by 35% (11). This association can be attributed to the additional physical stress on the spinal structure caused by weight gain, leading to an increased load on LBP. Essentially, an increase in body weight exacerbates the mechanical burden on the spine, thereby increasing the likelihood of developing and exacerbating lumbar disc herniation. However, the specific impact of high BMI on LBP is still inadequately explored, particularly concerning demographic variables such as the sociodemographic index (SDI), age, and sex. The GBD study is an extensive epidemiological research initiative aimed at systematically assessing the impact of various diseases, injuries, and risk factors on global health (12). Through the analysis of mortality rates, morbidity, and disability associated with numerous conditions, the GBD project provides a comprehensive health overview for countries and globally.

To further elucidate the relationship between high BMI and the global burden of LBP, we conducted an in-depth analysis of GBD data from 2021. This study investigates the temporal trends of LBP burden from 1990 to 2021, both globally and nationally, while also forecasting the impact of high BMI on the future burden of LBP over the next 15 years. Our findings aim to contribute further evidence for the implementation of effective policies and strategies to prevent and mitigate the increasing burden of LBP in the years to come.

## Materials and methods

### Data source

The 2021 Global Burden of Disease, Injury, and Risk Factor Study, conducted by the Institute for Health Metrics and Evaluation (IHME) (https://www.healthdata.org/data-tools-practices/interactive-data-visuals), constitutes the most comprehensive and detailed analysis of disease, injury, and risk factors on a global scale. The data generated from this study is openly accessible and publicly available, thereby establishing a free database. This database offers estimates of the global burden associated with 371 diseases and injuries, as well as 88 risk factors, across 204 countries and territories from 1990 to 2021. To extract the source data for our study, we accessed the official GBD 2021 data platform and utilized the “GBD Results Tool” to navigate to the data retrieval page. Within the “GBD Estimate” section, we selected “risk factor” to focus on specific risk factors contributing to disease burden. In the “Measure” section, we chose “Disability-Adjusted Life Years (DALYs)” to quantify the overall health impact. For the “Risk” category, we specified “high body mass index” as the risk factor of interest. In the “Cause” section, we selected “low back pain” to target the specific health outcome. By clicking “Search,” we could filter and retrieve the raw data pertinent to our study. Alternatively, we could click “Download” to obtain the data in a downloadable format. This systematic approach ensured that we received the precise data needed to analyze the impact of high body mass index on low back pain, as measured by DALYs, providing a robust foundation for our subsequent analysis and interpretation of the disease burden.

### Risk factors

The distribution of exposure for each risk factor was summarized using a summary exposure value (SEV). This metric compares the distribution of excess risk, adjusted by the exposure level, to a hypothetical population where all individuals are subjected to the maximum level of risk.

### Statistical analyses

To quantify the temporal patterns and evaluate trends in the age-standardized rate (ASR) of Disability-Adjusted Life Years (DALYs), this study employed the Average Annual Percentage Change (AAPC) to analyze the longitudinal trends in LBP. The ASR per 100,000 individuals is determined using the following formula:


$$\begin{array}{l} \text{ASR}=\frac{\sum_{\text{i}=1}^{\text{A}}{\text{a}}_{\text{i}}{\text{w}}_{\text{i}}}{\sum_{\text{i}=1}^{\text{A}}{\text{w}}_{\text{i}}}\times 100,000 \end{array}$$

where *a*_i_ denotes the ith age class and the number of persons (or weight), while (*w*_i_) in the same age subgroup *i* of the chosen reference standard population, *A*: number of age groups.

The Joinpoint regression model is a widely used statistical method for analyzing temporal trends in morbidity and mortality, particularly for chronic diseases such as cancer. In this study, Joinpoint Regression Analysis (JRA) was applied to identify significant changes in long-term trends.

Before modeling, we confirmed that the trend over the study period was non-uniform, justifying the application of Joinpoint modeling. The normality of the data distribution was assessed using the Shapiro-Wilk test, and the sample size was deemed sufficient to ensure the asymptotic normality of the parameter estimates. The fundamental principle of this regression model involves segmenting the long-term trend of morbidity or mortality in a time series into multiple segments at identified inflection points, referred to as Joinpoints. Key outcome indicators of the Joinpoint model include the Annual Percent Change (APC), the Average Annual Percent Change (AAPC), and the corresponding 95% Confidence Intervals (CI). The Joinpoint regression methodology employs ratios as inputs to detect years with significant trend changes and to compute the APC in these ratios between identified trend change points.

The APC is estimated using the following model:


$$\begin{array}{l} \log\left(Y_{X}\right)=b_{0}+b_{1}x\; \end{array}$$

where log (*Y*_x_) represents the natural logarithm of the ratio in year *x*.

The ratio is subsequently calculated from year *x* to year *x* + *b*_1_*X*, with log (*Y*_x_) denoting the natural logarithm of the ratio. Consequently, the APC from year *x* to year *x* + 1 is determined as follows:


$$\begin{array}{r} APC=\frac{e^{b_{0}+b_{1}x}\cdot e^{b_{1}}-e^{b_{0}+b_{1}x}}{e^{b_{0}+b_{1}x}}\times 100=(e^{b_{1}}-1)\times 100\\ AAPC=(e^{\frac{\sum w_{i}\beta_{i}}{\sum w_{i}}}-1)\times 100 \end{array}$$

*b*_i_ represents the slope coefficient of the segments in the specified yearly range, and *w*_i_ denotes their lengths. The ASR is deemed to trend upward if either the estimated annual percentage changes (EAPC) or AAPC and their 95% CI lower bounds are positive. Conversely, it trends downward if either the EAPC or AAPC and their upper 95% CI bounds are negative. If neither condition is satisfied, the age-standardized rate is considered stable.

Additionally, we conducted an analysis of the risk attribution for Disability-Adjusted Life Years (DALYs) rates per 100,000 individuals. This analysis utilized 95% uncertainty intervals (UIs), specifically the 2.5th and 97.5th percentiles, for both men and women across various SDI regions from 1990 to 2021. The SDI serves as an indicator of a country's per capita income distribution, average years of schooling, and female fertility rate for females under 25 years of age, thereby reflecting the overall level of development. Countries and regions are categorized into five quintiles based on their SDI: low, low-medium, medium, medium-high, and high.

The autoregressive integrated moving average (ARIMA) model is employed to forecast future outcomes based on time series derived from historical data and is extensively utilized for analyzing demographic and epidemiological trends in diseases. In this study, we developed an ARIMA model utilizing data from GBD 2021, where stationarity was achieved through appropriate differencing and confirmed using Augmented Dickey-Fuller (*p* < 0.01) and Kwiatkowski-Phillips-Schmidt-Shin (KPSS) (*p* > 0.05) tests. The optimal forecasting model was identified by selecting the one with the lowest Akaike Information Criterion (AIC) through the use of the “auto.arima()” function. The model's validity was assessed via a white noise test, where a residual test result of *p* > 0.05 indicated that the model successfully passed the white noise assessment. To enhance prediction accuracy, we complement ARIMA with a Bayesian age-period-cohort (BAPC) model, which is implemented by Markov chain Monte Carlo (MCMC) sampling in R (R-INLA package). The BAPC model takes into account the dynamics of age-specific risk, temporal trends, and birth cohort effects, thus addressing possible ARIMA. The BAPC framework takes into account age-specific risk dynamics, time trends, and birth cohort effects, thereby addressing the non-linear limitations that ARIMA may have.

All statistical analyses were conducted utilizing the “ggplot2” package within the R software environment (Version 4.4.1, R Core Team). Join-point analysis was run in the Joinpoint Regression Software (Version 5.1.0, Statistical Research and Applications Branch, National Cancer Institute, USA).

## Results

### The effect of high BMI on LBP burden

**Figure 1:**
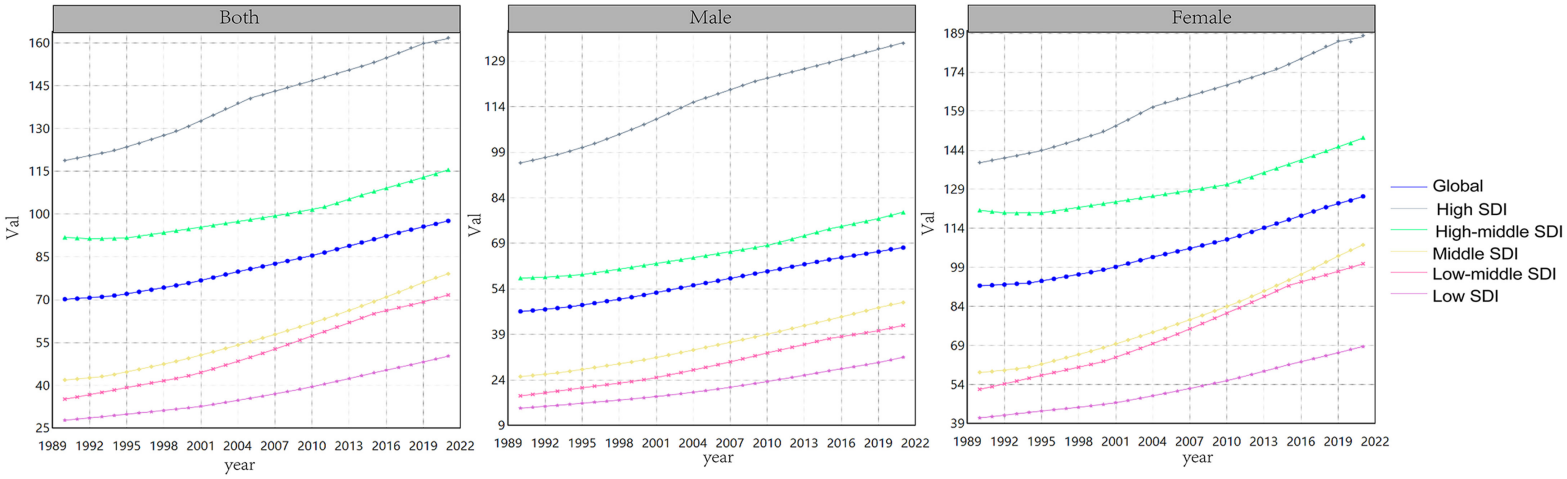
The age-standardized rate of Disability-Adjusted Life Years (DALYs) attributable to low back pain, stratified by high BMI, from 1990 to 2021. DALYs denotes the Disability-Adjusted Life Year, while high BMI represents the high BMI.

To evaluate the magnitude and direction of the trend in DALYs, a Joinpoint Regression Analysis (JRA) was performed. The findings, illustrated in Figure 1, Table 1, indicate that the impact of high BMI on low back pain increased annually from 1990 to 2021 (*p* < 0.001).

**Table 1:** Temporal trends in DALYs rates (per 100,000 population) attributable to high BMI across various SDI regions from 1990 to 2021.

**Socio-demographic index (SDI)**	**Year**	**High body-mass index**
Global DALY (95% CI)	1990–2000	0.08 (0.11–0.05)
	2000–2010	0.13 (0.14–0.11)
	2010–2021	0.14 (0.16–0.12)
Low SDI DALY (95% CI)	1990–2000	0.16 (0.2–0.11)
	2000–2010	0.23 (0.28–0.18)
	2010–2021	0.27 (0.32–0.22)
Low-middle SDI DALY (95% CI)	1990–2000	0.23 (0.28–0.18)
	2000–2010	0.32 (0.37–0.27)
	2010–2021	0.25 (0.3–0.21)
Middle SDI DALY (95% CI)	1990–2000	0.18 (0.22–0.13)
	2000–2010	0.25 (0.28–0.21)
	2010–2021	0.28 (0.31–0.22)
High-middle SDI DALY (95% CI)	1990–2000	0.03 (0.06–0)
	2000–2010	0.07 (0.09–0.05)
	2010–2021	0.14 (0.17–0.09)
High SDI DALY (95% CI)	1990–2000	0.1 (0.15–0.06)
	2000–2010	0.12 (0.15–0.1)
	2010–2021	0.1 (0.13–0.08)

The influence of high BMI on the burden of LBP across various genders, age groups, and SDI regions. SDI, sociodemographic index; CI, confidence interval; DALYs, Disability-Adjusted Life Years; DALYs per 100,000.

Additionally, regions with high SDI regions (HSDI), indicative of economic development, exhibited a significantly greater burden of disease. The analysis also revealed that the burden of disease was higher in females compared to males. Between 1990 and 2010, age-standardized DALYs rates attributable to high BMI factors were highest in low and middle-SDI regions, with factors of 0.23 (95% CI: 0.28–0.18) and 0.32 (95% CI: 0.37–0.27), respectively.

The GBD 2021 study indicates that in 2021, the DALYs attributable to LBP due to high BMI totaled 8,363,759.33 (95% CI: 8,403,066.54 to 17,424,821.68). The APC in DALYs from 1990 to 2021 was 1.71 (95% CI: 1.57–1.83), reflecting a consistent increase in the burden of LBP associated with high BMI over the past three decades (Table 2). Further analysis across five socio-demographic index (SDI) regions revealed that high SDI regions accounted for the highest DALYs due to LBP attributable to high BMI, at 2,478,626.07 (95% CI: 2,492,112.24–5,091,840.29). In contrast, low SDI regions experienced the lowest burden, with DALYs at 356,409.63 (95% CI: 35,002.73–729,956.95). These findings highlight the substantial global impact of high BMI on LBP, with significant regional disparities in disease burden, largely corresponding to levels of economic development.

**Table 2:** Disability-Adjusted Life Years (DALYs) and age-standardized DALYs rate attributed to LBP for high BMI in 1990 and 2021.

**Location**	**DALYs**			**ASDR (per 100,000 population)**		
	**1990 (95% UI)**	**2021**	**APC**	**1990**	**2021**	**APC**
Global	3,086,573.08 (312,559.12, 6,484,427.06)	8,363,759.33 (840,306.54, 17,424,821.68)	1.71 (1.57, 1.83)	70.22 (7.14, 146.48)	97.66 (9.78, 204)	0.39 (0.32, 0.45)
High SDI	1,218,421.82 (119,569, 2,549,631.15)	2,478,626.07 (249,211.24, 5,091,840.29)	1.03 (0.88, 1.19)	118.84 (11.6, 248.8)	161.8 (16, 332.59)	0.36 (0.26, 0.46)
High-middle SDI	956,976.62 (96,319.02, 2,014,411.5)	2,086,188.17 (212,179.44, 4,263,358.45)	1.18 (0.97, 1.3)	91.9 (9.24, 192)	115.59 (11.61, 238)	0.26 (0.16, 0.31)
Low SDI	82,343.43 (9,051.79, 165,429.39)	356,409.63 (35,002.73, 729,956.95)	3.33 (2.89, 3.6)	27.81 (3.09, 55.76)	50.3 (5.07, 103.18)	0.81 (0.63, 0.92)
Low-middle SDI	276,060.91 (29,215.8, 573,856.68)	1,227,064.64 (122,732.05, 2,554,600.6)	3.44 (3.06, 3.73)	35.21 (3.78, 72.71)	71.69 (7.24, 148.95)	1.04 (0.86, 1.16)
Middle SDI	547,306.42 (57,862.32, 1,153,131.65)	2,205,381.99 (219,659.9, 4,678,252.07)	3.03 (2.7, 3.28)	41.92 (4.49, 87.56)	79.1 (7.86, 168.24)	0.89 (0.74, 0.99)

SDI, sociodemographic index; UI, uncertainty interval; APC, annual percent change; DALYs, Disability-Adjusted Life Years; ASDR, age-standardized DALYs rate.

**Figure 2:**
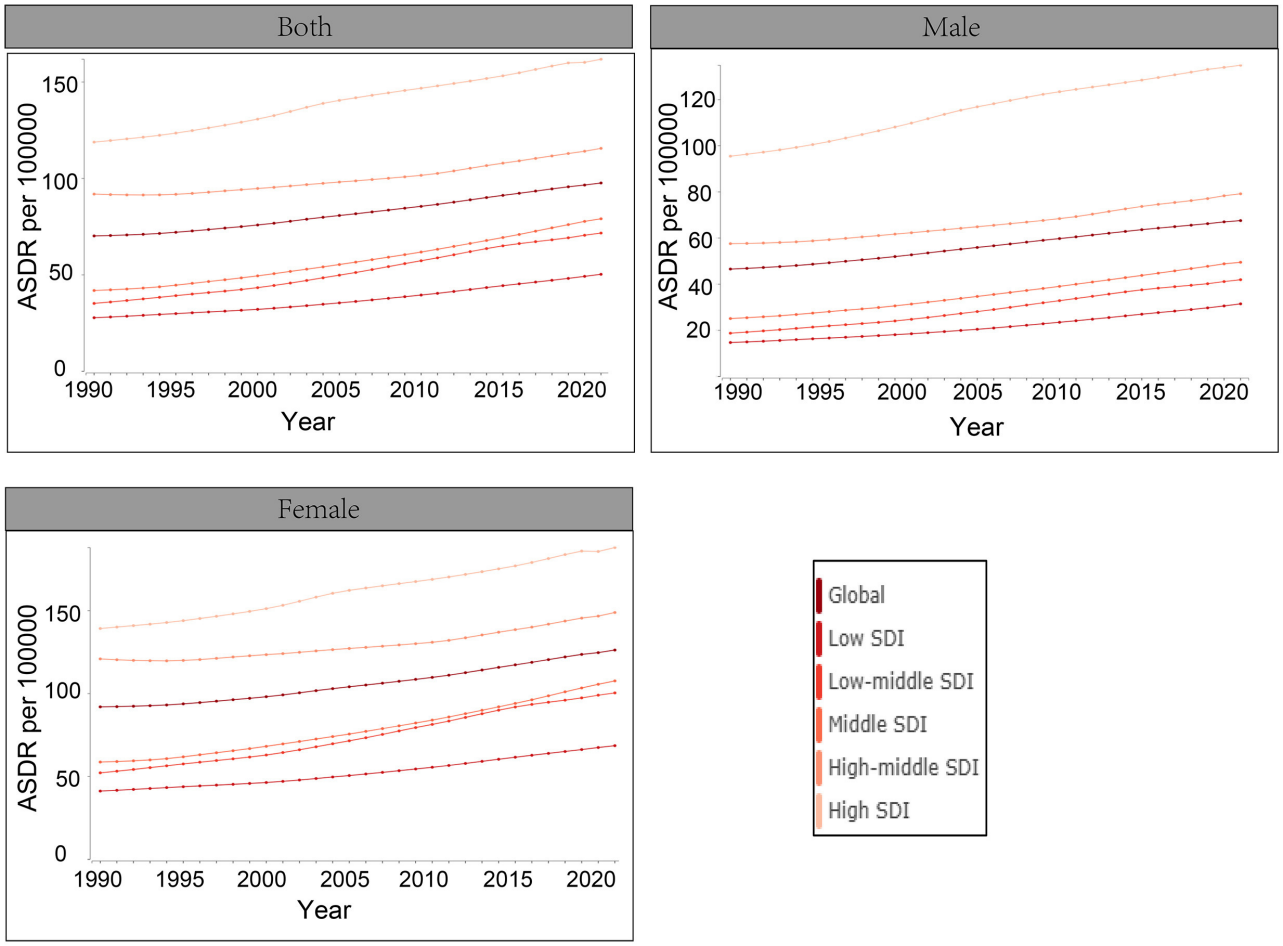
Age-standardized rates of Disability-Adjusted Life Years (DALYs) attributable to high BMI from 1990 to 2021.

Due to the absence of mortality data for LBP, this study exclusively reports age-standardized DALYs data for high BMI, as illustrated in Figure 2. The findings indicate a consistent year-over-year increase in the global burden of high BMI on LBP from 1990 to 2021. Notably, the global burden of high BMI on LBP was more pronounced among females in regions with HSDI scores.

**Figure 3:**
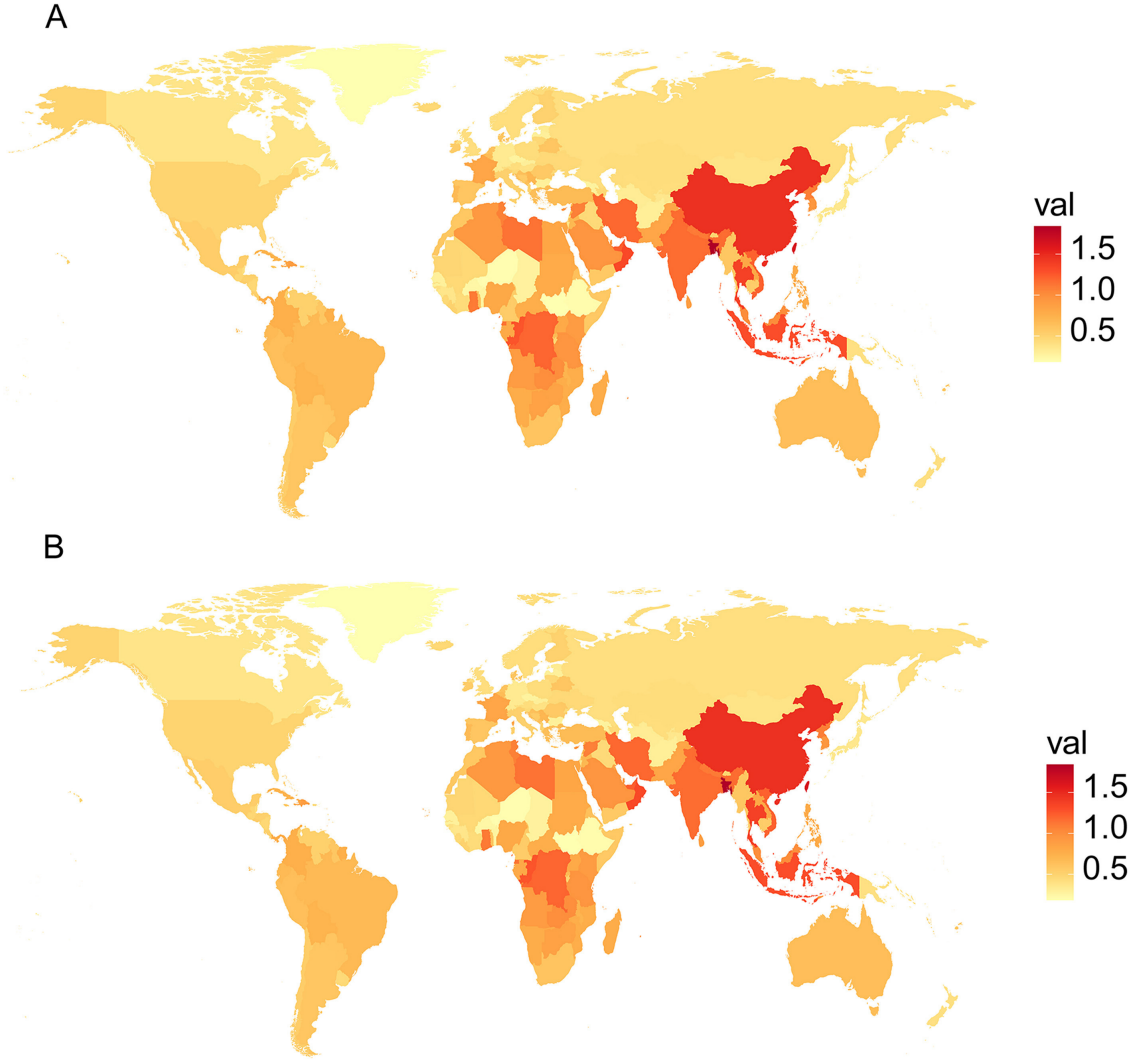
Percentage change in age-standardized SEV for males **(A)** and females **(B)** with high body mass index (BMI) across 204 countries from 1990 to 2021.

The percentage change in age-standardized summary exposure value (SEV) for females and males with high BMI across 204 countries from 1990 to 2021 is illustrated in Figure 3. Variations in the burden of LBP are correlated with differences in sex and geographic region, revealing disparities in SEV both between regions when analyzed by a single sex and between females and males when compared within the same region. From 1990 to 2021, some countries in Africa and Asia had shown the most significant changes in LBP caused by high BMI in both females and males, especially among females in China.

**Figure 4:**
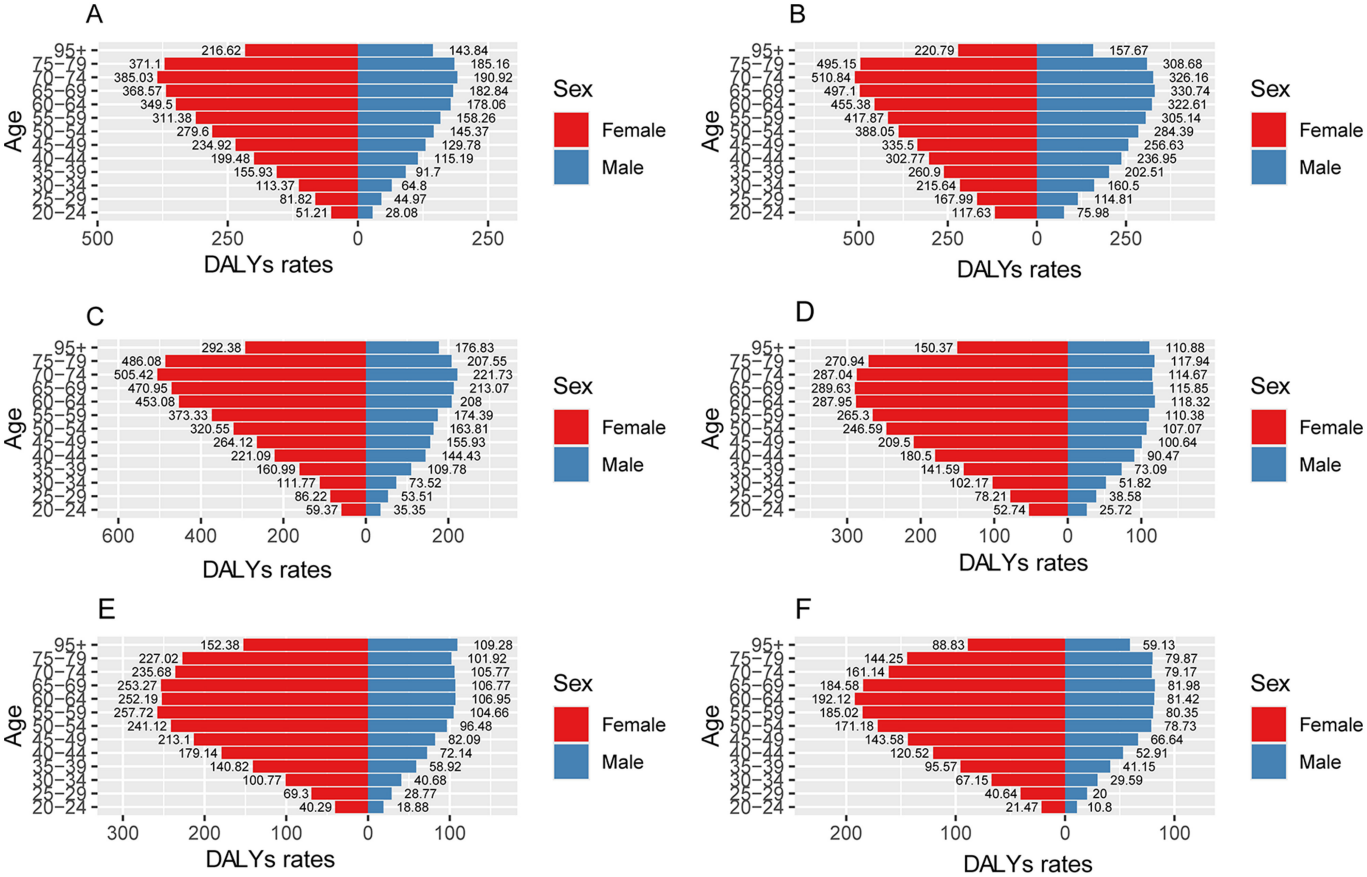
DALYs for SDI and gender in 2021. **(A)** Is global, **(B)** is high SDI, **(C)** is high-middle SDI, **(D)** is middle SDI, **(E)** is low-middle SDI, **(F)** is low SDI.

Two-sided bar charts were constructed utilizing age and gender groupings (Figure 4). The findings indicated that, within the same economic level, individuals of older age experience a greater burden of disease. Furthermore, within the same age group, females exhibit a higher burden of disease compared to males. In the 65–69 age group, females experienced a significantly higher burden of LBP attributed to BMI, with 368.57 DALYs compared to 182.84 DALYs in males. This trend persisted in the 70–74 age group, where DALYs were 385.03 for females and 190.92 for males. In contrast, the 20–24 age group had the lowest burden of LBP due to high BMI, with 51.21 DALYs for females and 28.08 DALYs for males. The prevalence of LBP was notably higher among individuals aged 70–74 years compared to other age groups.

**Figure 5:**
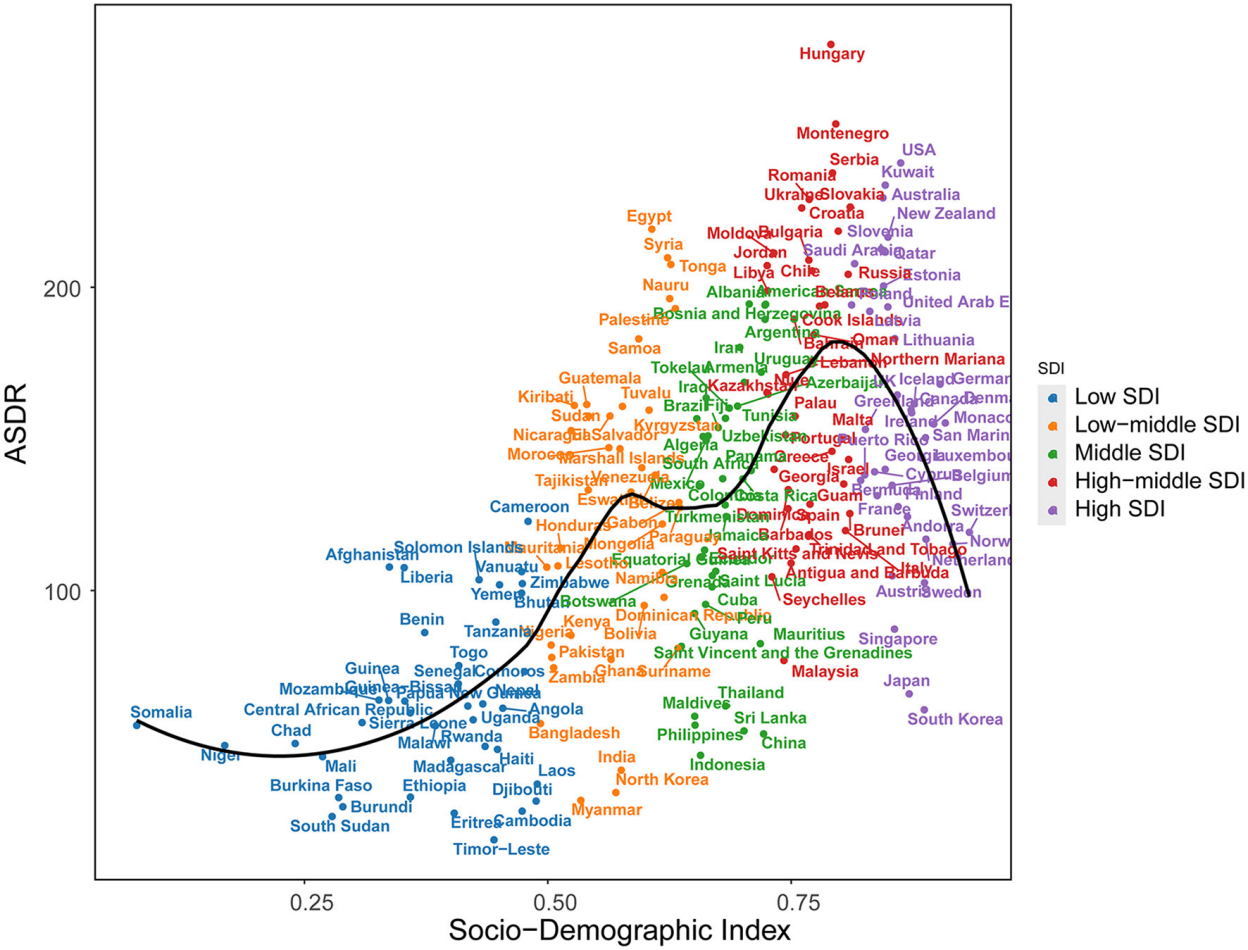
Age-standardized years DALYs of LBP by 204 countries and territories and SDI, 2021; expected values are shown as the black line. Each point shows observed Age-standardized years DALYs for specified country in 2021.

The relationship between age-standardized DALYs and SDI in 204 countries and regions in 2021 is shown in Figure 5. The findings indicate that the disease burden progressively escalates from low-economic regions (low SDI), such as Somalia, Niger, Chad, and Burkina Faso, to medium-high economic regions (high-middle SDI), including South Africa, Mexico, and Colombia. However, as economic development continues, a decline in disease burden is observed in high-income areas, such as the United States, Australia, and New Zealand.

### Projections for the future

**Figure 6:**
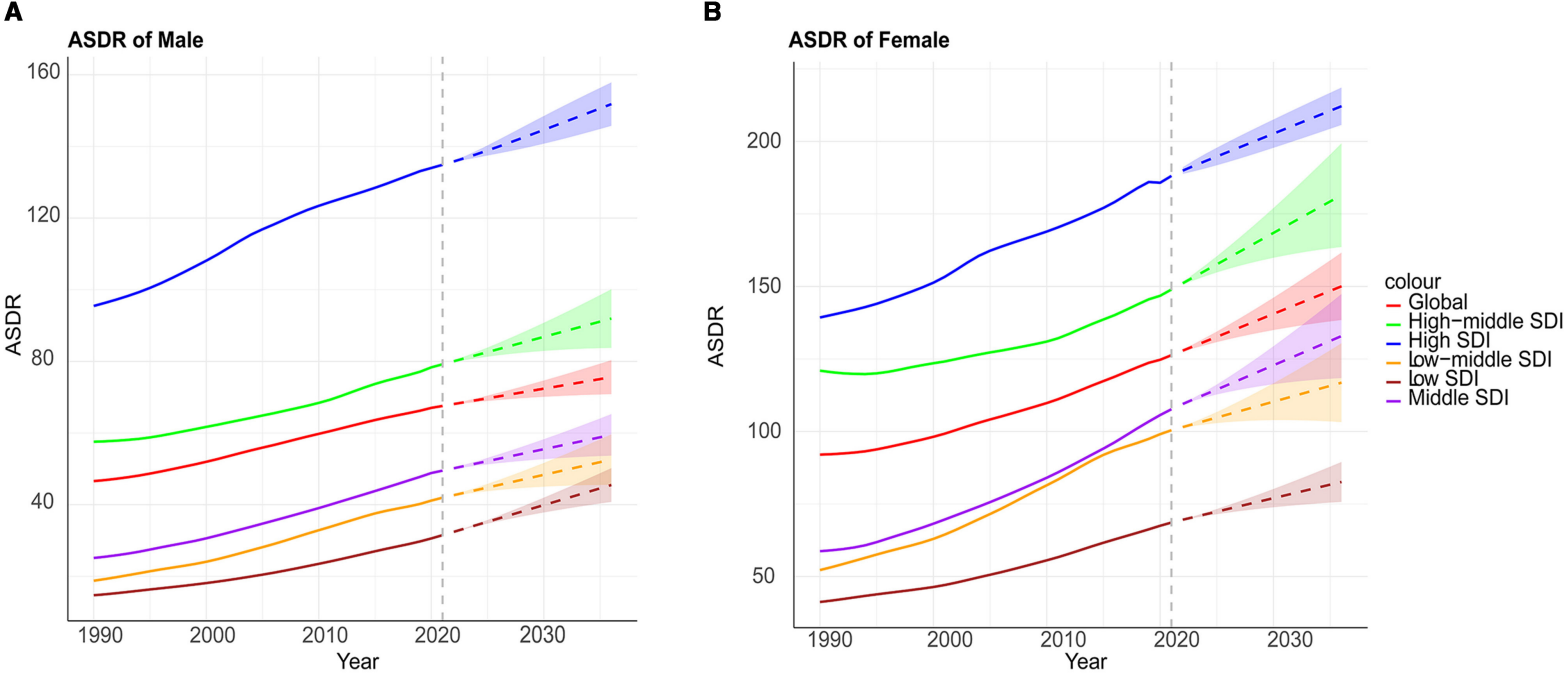
Projections of the global burden of disease attributable to LBP across SDI quintiles and by males **(A)** and females **(B)** are represented as high BMI. The *x*-axis denotes the year, while the *y*-axis represents DALYs per 100,000 population.

Utilizing age-standardized DALYs (ASDR) spanning from 1990 to 2021, the ARIMA model was used to project the trajectory of the global disease burden of high BMI over the next 15 years for different SDI regions associated with LBP (Figure 6). The global burden of LBP continues to rise, with the highest burden observed in regions with high SDI. Projections for 2036 indicate that the ASDR for LBP attributable to high BMI will be 72.6 per 100,000 for men and 150.0 per 100,000 for females. The ASDR for LBP attributable to high BMI by 2036 is projected to be higher in high SDI regions, with 151.8 per 100,000 for men and 212.1 per 100,000 for women, compared to lower rates in low SDI regions (45.5 per 100,000 for males and 82.6 per 100,000 for females). Women consistently experience a higher burden of LBP than men, with the burden in women in high SDI regions projected to be 1.4 times higher than in men.

**Figure 7:**
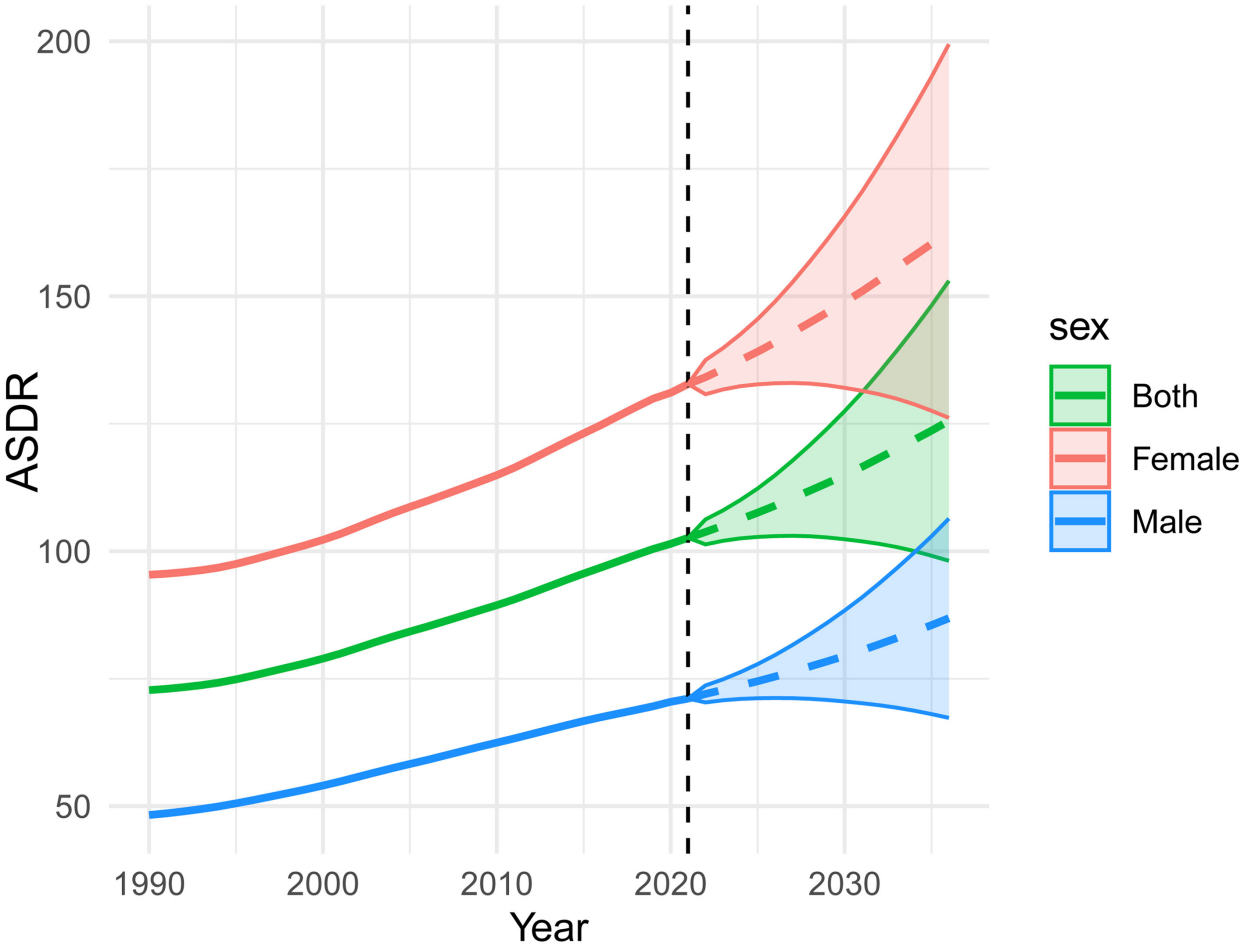
Future projections of LBP attributable to high BMI based on the BAPC model.

The BAPC model projections revealed a concerning upward trend in the global ASDR of LBP attributable to high BMI from 2022 to 2036. By 2036, the projected ASDR reached 125.57 per 100,000 population (95% UI: 98.11–153.03) in the overall population, with significant gender disparities observed, 86.86 per 100,000 population (67.32–106.39) for males and 162.79 per 100,000 population (126.15–199.43) for females (Figure 7).

## Discussion

A high BMI is often associated with a higher risk of several health conditions, including low back pain, cardiovascular diseases, type 2 diabetes, hypertension (13), sleep apnea (14), and certain cancers. Previous research from the GBD 2021 Low Back Pain Collaborators revealed that low back pain affected 619 million people globally in 2020, with a projection of 843 million prevalent cases by 2050 (1). A total of 38.8% (28.7–47.0) of YLDs were attributed to occupational factors, smoking, and high BMI. In this study, we found that the impact of high BMI on low LBP has shown a steady increase, with the global burden of LBP associated with high BMI also rising annually from 1990 to 2021. Some studies have shown that physical activity levels and body fat index have a significant impact on the fatty infiltration of the lumbar multifidus muscle, which is critical for the integrity and function of the lumbar spine. Specifically, there is a positive correlation between BMI and fatty infiltration of the lumbar multifidus muscle, indicating that higher BMI may be associated with increased fatty infiltration in this muscle (15). This, in turn, could potentially contribute to or exacerbate symptoms of low back pain. As we all know, disc degeneration also causes the symptom of low back pain. Previous studies revealed that there may be an association between the severity of disc degeneration and high BMI. Furthermore, the public and spine surgeon should emphasize the relationship between recurrence in patients undergoing single-level lumbar disc herniation surgery and high BMI (16).

Furthermore, our studies showed that regions with a high SDI, representing more developed economies, exhibit a significantly higher disease burden. It has also been observed that within regions of similar economic levels, the burden of LBP increases with age. This finding is consistent with previous research results. Chen et al. (17) found that developed and high-income countries had higher levels of age-standardized prevalence of LBP. Interestingly, in lower to middle-high SDI regions, the burden of LBP increases with economic growth. However, in high SDI regions, the burden of LBP decreases as economic development progresses. We discussed that while high BMI rates are increasing, high SDI regions often benefit from earlier diagnosis, better management of musculoskeletal disorders, and more widespread preventive healthcare services, which can partially offset the expected rise in LBP burden. Additionally, awareness campaigns and workplace ergonomics improvements may contribute to this trend.

Social factors, such as work conditions and lifestyle, play a significant role in the development of LBP (11, 18). Prolonged sitting, sedentary behavior, and physically demanding jobs increase strain on the lower back, raising the risk of LBP (19). People from lower socioeconomic backgrounds also face barriers to healthcare, which can worsen LBP outcomes. Environmental factors like urban living and air pollution may contribute indirectly by reducing physical activity and increasing stress (20). High SDI regions often have more opportunities for physical exercise and better urban planning, whereas rapid unplanned urbanization in low SDI areas may increase LBP risk factors. Similarly, disparities in healthcare accessibility greatly influence early detection and treatment outcomes. Future research should explore how these factors interact with BMI and other risks to provide a more comprehensive understanding of LBP and improve prevention strategies.

The burden of LBP attributed to high BMI shows significant variation across age groups. In older adults, particularly in the 70–74 age range, the burden of LBP is exacerbated due to the cumulative effects of aging, including degenerative changes in the spine, reduced physical activity, and decreased tissue repair capacity. In contrast, younger adults (e.g., 20–24 years), despite having a lower prevalence of degenerative spinal conditions, are still at risk of LBP due to the systemic inflammatory effects of obesity, which can lead to musculoskeletal pain even in the absence of structural damage. This age-related disparity in LBP burden highlights the need to consider not only BMI but also lifestyle factors such as physical activity levels and occupation, as they interact with BMI to influence the development of LBP. For instance, sedentary lifestyles and prolonged sitting, particularly common in younger working-age populations, may exacerbate the mechanical stress of obesity on the lumbar spine. Furthermore, socioeconomic factors may also play a role, with individuals from lower socioeconomic backgrounds facing greater barriers to accessing preventive healthcare and treatment for LBP, thus experiencing a higher burden.

Additionally, our series also analyzed the burden of LBP attributed to sex differences between 1990 and 2021 to reveal different trends and distribution characteristics of the burden. We found that females experience a greater disease burden than males within the same age group. In many different series of studies conducted in the past, poor sitting posture, smoking, aging, low levels of physical activity, and high BMI have been identified as potential risk factors for low back or spinal pain (21–24). However, these risk factors do not account for gender differences. Biological and social factors contribute to this gap. Biologically, women may be more prone to LBP due to differences in spinal structure (25), hormonal changes (e.g., during pregnancy and menopause), and a greater lumbar curvature. Socially, women are more likely to have jobs that involve prolonged sitting or physical labor, which increases back strain (26). Additionally, women often face barriers to healthcare, which can worsen LBP outcomes. Some studies have also indicated that gender may influence low back pain, particularly in terms of its classification and treatment. Ono et al. demonstrated that sex and gender play a role in analyzing disability and health-related quality of life (HRQOL) in patients with low back pain. Their findings suggested that females experience higher levels of disability, while HRQOL is significantly affected by varying gender role orientations (27). And a cross-sectional study based on a population survey highlighted that factors such as older age, lower education levels, hypertension, and smoking were linked to low back pain among men (28). In women, occupational and ergonomic factors were more strongly associated with the condition. Additionally, marital status was found to be a common factor associated with low back pain in both genders. To address these gender differences, we recommend gender-specific policies. For women, improving workplace ergonomics, such as adjustable chairs and proper lifting techniques, and increasing access to preventive care are essential. Programs promoting physical activity and weight management, particularly during pregnancy and menopause, should also be prioritized. For men, policies should focus on reducing physical strain in male-dominated occupations (e.g., construction) by improving safety measures and ergonomics. Both genders would benefit from better access to preventive healthcare, especially those in lower-income groups. Based on data from 1990 to 2021, we utilized the ARIMA model to predict the trend of high BMI on global burden of LBP over the next 15 years, and the results show that the global burden of disease will continue to rise in both genders. Given the projected increase in the global burden of LBP among both men and women by 2040, coordinated policy actions should be taken to mitigate this escalating health issue. Considering the consistent association between high BMI and the incidence of LBP, optimizing high BMI should be prioritized. Shifts in disease attribution influence the direction of health promotion efforts. Our research highlights the importance of reducing high BMI, prompting policymakers to allocate resources more effectively for early diagnosis and mitigation of this modifiable risk factor for LBP. Preventing and improving unhealthy high BMI through dietary adjustments, appropriate daily exercise, and the proper use of medications can help reduce the risk of LBP. The ARIMA model assumes linear growth over time, which may not capture sudden changes, such as those caused by global pandemics. Although widely used for long-term forecasting, its assumptions of linearity and stability limit its ability to account for non-linear fluctuations. Future studies should consider more flexible models, such as machine learning or state-space models, to better capture abrupt shifts in health data (29, 30). Incorporating real-time data and external factors, like public health interventions, can improve model accuracy and robustness.

Of course, there are several limitations to our studies worth acknowledging. Firstly, the GBD database relies on data from different countries and regions, and the quality and completeness of these data may vary. Some developing countries may lack well-established health monitoring systems, resulting in incomplete or inconsistent data. Secondly, in cases of missing or incomplete data, the GBD project relies on statistical models for estimation and projection. These models are based on assumptions that may not accurately reflect the actual situation. Thirdly, due to the time required for data collection and analysis, the GBD database typically reflects health conditions from previous years rather than real-time data. For rapidly evolving health issues, such as emerging diseases or pandemics, the GBD may not be able to provide timely and accurate burden assessments. In addition, the GBD uses complex statistical models for estimation, and these models rely on a series of assumptions, such as linear growth or stable trends in population health data. In some cases, these assumptions may be inaccurate, affecting the precision of the results. Last but not least, although the GBD considers various health risk factors, it may be limited in capturing the broader impacts of social, economic, and environmental factors on health. Complex factors such as poverty, education level, and climate change often have long-term health effects that are difficult to quantify. To improve the accuracy and timeliness of future research, we recommend strengthening global health surveillance systems, particularly in resource-limited areas. Additionally, interdisciplinary research should be encouraged to gain a more comprehensive understanding of the social, economic, and environmental determinants of health.

## Conclusion

In summary, the burden of low back pain continues to escalate globally, influenced by a myriad of interconnected factors. In the past 30 years, the global burden of LBP attributable to high BMI has increased, with a disproportionately higher burden observed among women and in low SDI regions. Furthermore, this burden is predicted to continue rising over the next 15 years. This introduction sets the stage for a comprehensive analysis of how high BMI serves as a significant risk factor for LBP and its implications for public health. By leveraging GBD data and analyzing trends, this research aspires to provide valuable insights that can inform future interventions to address and reduce the incidence of LBP, ultimately improving quality of life and healthcare outcomes for affected individuals.

## Data Availability

The original contributions presented in the study are included in the article/supplementary material, further inquiries can be directed to the corresponding authors.
